# Complex Functional Posttraumatic Shoulder Reconstruction Using Shoulder Arthroplasty and a Pedicled Innervated Latissimus Dorsi Flap—A Case Report and Literature Review

**DOI:** 10.1111/os.13575

**Published:** 2022-11-21

**Authors:** Olimpiu Bota, Adrian Dragu, Florian Bönke, Eric Tille, Feras Taqatqeh, Jörg Nowotny

**Affiliations:** ^1^ University Center for Orthopedics, Trauma and Plastic Surgery, Faculty of Medicine Carl Gustav Carus TU Dresden Dresden Germany

**Keywords:** Case report, Humeral hemiarthroplasty, Innervated latissimus dorsi flap, Shoulder reconstruction, Soft tissue coverage

## Abstract

**Background:**

The shoulder joint is one of the most freely movable joints in the human body and has therefore high importance for upper limb functionality. Several techniques have been developed to replace the glenohumeral joint including humeral hemiarthroplasty, anatomical total shoulder arthroplasty, and reverse total shoulder arthroplasty, depending on the underlying pathology. For the soft tissue reconstruction, the innervated latissimus dorsi musculocutaneous flap is a reliable solution flap in shoulder and arm reconstruction.

**Case presentation:**

We present the case of a 16‐year‐old male patient with a complete destruction of the shoulder joint and soft tissues after ballistic trauma. We performed the reconstruction of the shoulder joint using a humeral hemiarthroplasty with a mesh fixation to the remaining glenoid. The soft tissue coverage and the restoration of the deltoid muscle function were insured with a pedicled innervated latissimus dorsi musculocutaneous flap. One year postoperatively, the patient showed a good function of the shoulder joint with an excellent aesthetical result and no pain.

**Conclusion:**

The pedicled latissimus dorsi musculocutaneous flap can safely restore the shoulder function, while the humeral hemiarthroplasty with mesh fixation can be a reliable solution for the reconstruction of a completely destructed shoulder joint.

## Graphical abstract

The completely destroyed shoulder joint can be functionally reconstructed using humeral hemiarthroplasty with mesh fixation and pedicled innervated latissimus dorsi flap.

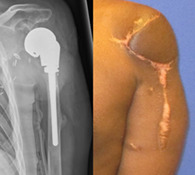

## Introduction

The shoulder joint is a synovial, bone‐and‐socket joint, with three degrees of movement. Being one of the most freely movable joints in the human body, it has high importance for the functionality of the upper limb. The movements of the glenohumeral joint are insured by a multitude of muscles (especially the rotator cuff). The three‐part planar deltoid muscle is most remarkable and is through its anterior, middle, and posterior proportion is responsible for the flexion and internal rotation (anterior part), the abduction (middle part), and the extension and external rotation (posterior part) of the shoulder. It also covers the glenohumeral joint and confers the aesthetical aspect of the shoulder.^1^ In the case of no reconstruction possibilities, several techniques have been developed to replace the glenohumeral joint including humeral hemiarthroplasty, anatomical total shoulder arthroplasty, and reverse total shoulder arthroplasty, depending on the underlying pathology.^2^

The pedicled latissimus dorsi musculocutaneous flap (LDMF) is a workhorse flap in the thorax, neck and upper arm regions. It offers bulky soft tissue coverage with the possibility of functional reconstruction, when harvested as an innervated flap.^3^, ^4^ The use of LDMF to functionally reconstruct the shoulder movement with the addition of a hemiarthroplasty has not been to our knowledge published before.

## Case Presentation

**Fig. 1:**
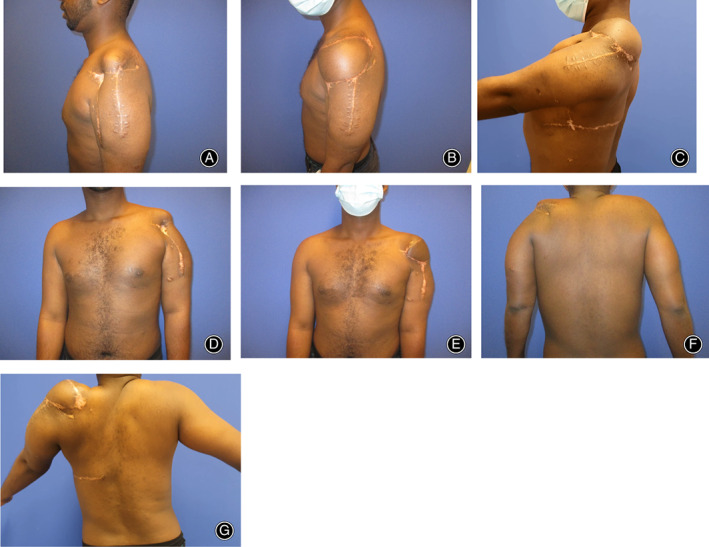
(A) Preoperative clinical photos from the side. (B) Postoperative clinical photo from the side after 1 year. (C) Postoperative clinical photo with maximal arm flexion after 1 year. (D) Preoperative clinical photo from the front. (E) Postoperative clinical photo from the front after 1 year. (F) Preoperative clinical photo from the back. (G) Postoperative clinical photo from the back with maximal arm abduction after 1 year

**Fig. 2:**
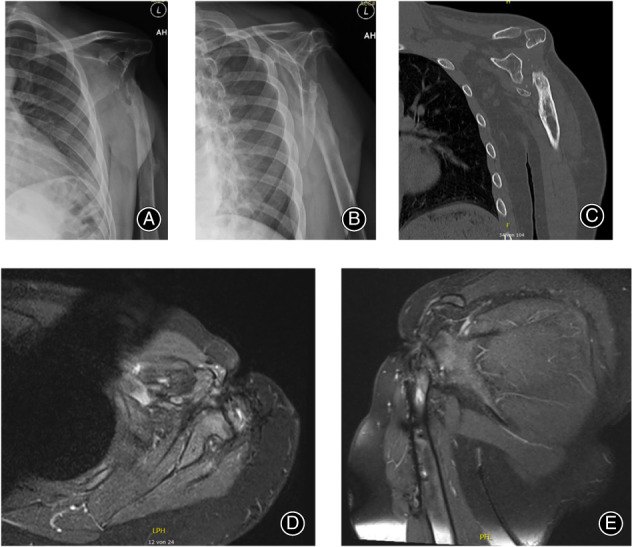
(A) and (B) Preoperative X‐ray in two planes with proof of progressive destruction of the glenohumeral joint. (C) Coronal slice of the CT. (D) and (E) Axial and coronal MRI with proof of insufficient rotator cuff and advanced soft tissue destruction

A 16‐year‐old male patient presented at our clinic with complete destruction of the left shoulder joint, the surrounding muscles including deltoid, coracobrachialis and long biceps tendon and an unstable scar covering the remaining of the shoulder girdle (Fig. 1A,D,F). Before fleeing his native country, he had suffered at the age of 12 years old a ballistic trauma to his left shoulder in a war context. At the consultation, he showed an almost completely immobile shoulder joint with a deformed, scarred contour in this area, and debilitating chronic pain. The appearance of the shoulder region was causing the patient supplemental psychological distress in the context of the posttraumatic stress disorder (PTSD). The X‐rays showed a complete destruction of the proximal humerus without proof of a humeral head, as well as a significant deformed glenoid (Fig. 2A,B). CT and MRI scans were also performed, showing no active signs of osteomyelitis, no sufficient rotator cuff and a persistent latissimus dorsi muscle (LD) with the thoracodorsal artery (Fig. 2C–E). The external soft tissue coverage of the shoulder was replaced by scar tissue. After the complete diagnosis, a therapeutical plan was then established. The first step was beginning the psychotherapeutical treatment of PTSD. Finally, the surgical procedure was planned, in order to restore the bony skeleton, the shoulder joint, the motor function, the soft tissue coverage, and last but not least the aesthetics of the region.

### Surgical Procedure

**Fig. 3:**
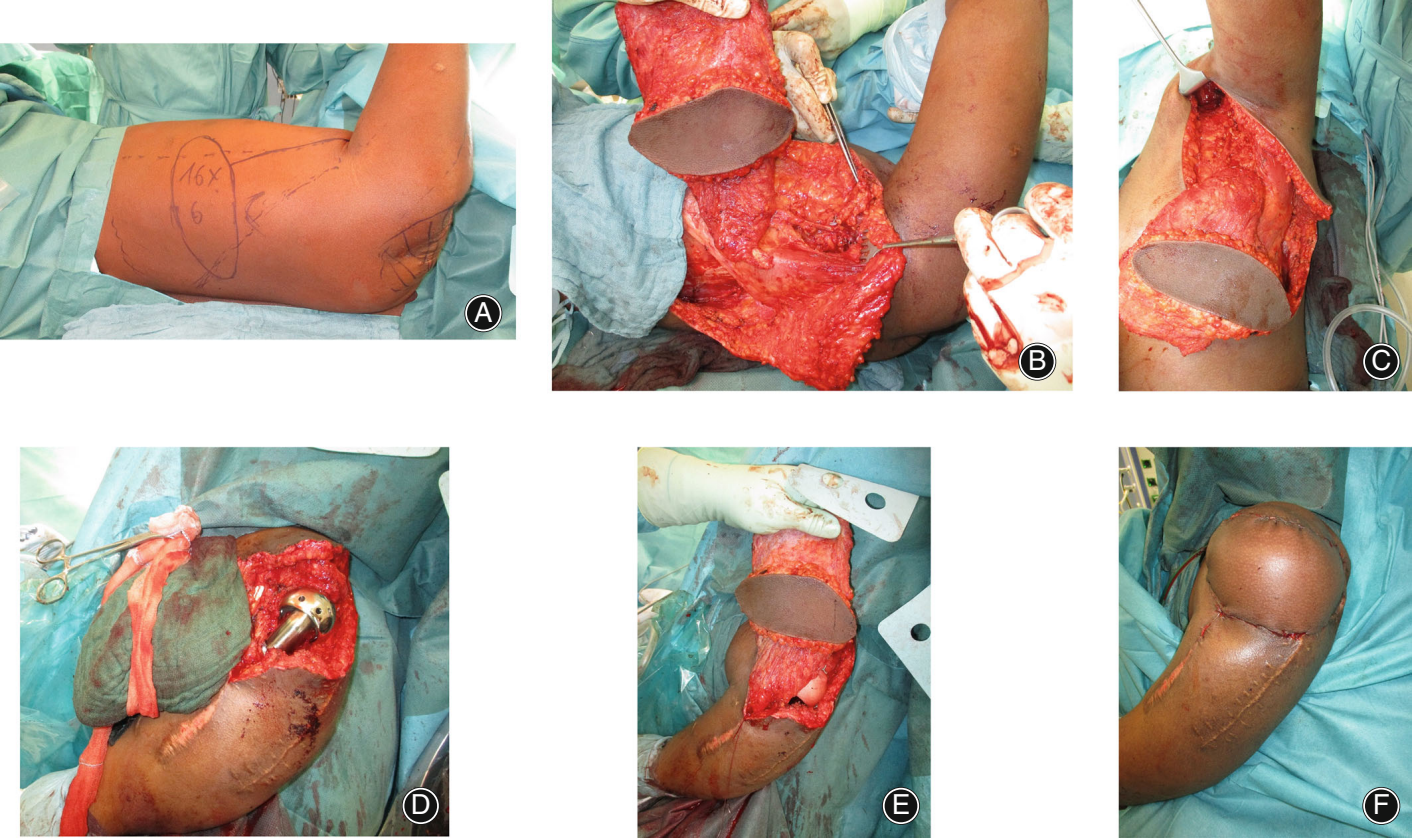
(A) Intraoperative flap planning with patient in lateral decubitus position. (B) Elevated flap with the ready dissected thoracodorsal pedicle. (C) Subcutaneous tunnel prepared for flap transfer. (D) Installed humeral prosthesis, flap safely stored in a cloth. (E) Flap placed in position, underneath the humeral prosthesis is fixated in the Gore‐Tex mesh. (F) Final intraoperative result

Approximately 6 months after beginning the outpatient psychotherapy, he was admitted to our institution and the surgery was performed under general anesthesia. The procedure began in right lateral decubitus position with the left arm in 90° of abduction. The anterior border of the LD was identified and a transverse elliptical skin island 16 × 6 cm was designed on the muscle 15 cm caudal from the axillary fold (Fig. 3A). The dissection began on the anterior border of the LD, in order to identify the thoracodorsal neurovascular bundle and the detached tendon from the humeral insertion. After that, the flap was elevated as described by Bota *et al*.^4^ (Fig. 3B). A subcutaneous tunnel was prepared anteriorly and the scar tissue in the shoulder area was removed (Fig. 3C). The flap was passed through the tunnel and temporarily positioned in the defect. Care was taken to avoid kinking or tensioning the pedicle. The donor site was then closed and the patient was moved to a beach‐chair position.

At this point, the bone was exposed and, in regard to the initial septic trauma, thoroughly debrided. The scared proximal humeral shaft was resected and the medullary cavity was drilled out. Several tissue samples were sent for microbiological and histopathological examination. A hemiarthroplasty by using a modular segmental revision system with extended articular surface head, Comprehensive (Zimmer Biomet, Warsaw, IN, USA) was press‐fit implanted in the humeral shaft (Fig. 3D). Due to the deformity of the glenoid, the humeral head was centered to the glenoid using an embracing Gore‐Tex mesh (W. L. Gore & Associates, Newark, DE, USA). Finally, the LD was brought into place as to restore the deltoid muscle and replace the soft tissues (Fig. 3E). The tendon was inserted using a bone anchor in the humerus at the level of the deltoid tuberosity. Cranially, the LD origin was spread out and sutured to the fascia of the trapezius muscle. Finally, the skin was directly closed, using the skin island to replace the scar tissue (Fig. 3F).

### Postoperative Care

Postoperatively, the left arm was immobilized in 45° of abduction for 3 weeks, after which occupational—and physiotherapy was begun. The flap healed without complications. The microbiological investigation of the humeral bone samples showed growth of Enterobacter cloacae and Staphylococcus epidermidis. A 6‐week regimen of Carbapenems was begun according to the antibiogram.

### Outcomes

**Fig. 4:**
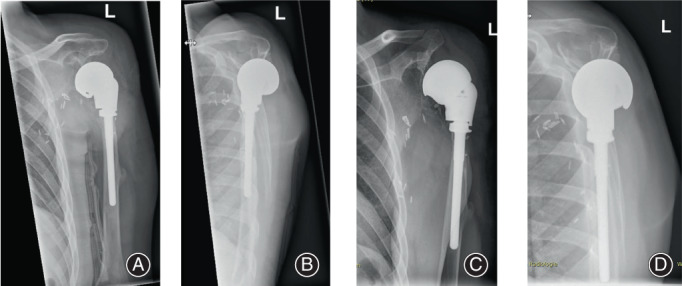
Postoperative X‐ray of the shoulder joint in two planes with proof of regular centering of the shoulder. (A) and (B) 3 months postoperative. (C) and (D) 12 months postoperative

At follow‐up 1 year after the operation, the scars were inconspicuous and with clothing there was a relative symmetry with the contralateral healthy side. X‐ray follow‐up after 3 and 12 months shows a correct implant position without loosening and correct centering of the shoulder joint (Fig. 4A–D). The range of motion of the shoulder for adduction/abduction was 30/0/40° and for anteversion/retroversion 75/0/30°. The patient reported high satisfaction with the aesthetical and functional result without pain, the PTSD being in regression.

This study was approved by the Ethics Committee of TU Dresden (EK377102014).

## Discussion

In this article, we present the complex functional reconstruction of bone, joint, muscle, and soft tissue of a progressive destroyed shoulder after ballistic trauma. The loss of the glenohumeral joint, the proximal humerus and the adjacent soft tissues could be reconstructed in a single surgery, restoring the form, the function and the aesthetics of the region appropriately, all at once.

A special challenge in achieving the reconstructive goal was the restoration of the extensively destroyed glenohumeral joint. For reconstruction of the shoulder joint, a modular revision hemiprosthesis was chosen in regard of the absence of a rotator cuff and sufficient deltoid muscle, which enables a more limited range of motion, than a reverse shoulder arthroplasty (RSA). However, RSA would require a sufficient glenoid bone augmentation of the present glenoidal neo‐cavitas and there would be a less calculable risk for dislocation due to the significant soft tissue damage.

In the literature, there are comparable challenges after oncological surgery with large bony and soft tissue defects, in which endoprosthetic reconstruction has proven its worth.^5^, ^6^, ^7^ Modern modular arthroplasty aims to restore the anatomic structure, contour of the shoulder and mobility that allows patients to perform fundamental activities that were not possible before.^8^ Reverse total shoulder arthroplasty should be primarily performed when both the deltoid muscle and axillary nerve are preserved to achieve the good results in terms of postoperative functionality.^9^, ^10^ Both conditions were not met in the present case and it is known, that the rate of failure is high after these complex operative interventions.^10^, ^11^, ^12^

Although our patient had no open wounds in the past 4 years, an unexpected positive culture from the humeral bone was detected. The potential explanation could be either a contamination of the probe, which is rather unlikely as the sample was originated from the bone drilling and it contained a multiresistant strain of Enterobacter cloacae, or as a possible persistence of dormant bacteria from the initial injury. We opted for a prolonged course of intravenous carbapenems according to the antibiogram as a measure of safety. The 2018 International Consensus Meeting on Orthopedic Infections concluded nevertheless that to date there was not enough evidence to support a universal standard of treatment in these cases, including antibiotics, reoperation or wait and see.^13^ A two‐stage approach could have been a safer alternative, with bone debridement, spacer placement, and flap coverage at the first surgery. Nevertheless, re‐elevating the healed flap could have led in this case to pedicle injury or loss of function and therefore a one‐step approach was chosen.

The pedicled LDMF is known to be a reliable flap for functional reconstruction of elbow flexion and soft tissue coverage of the upper arm.^14^ Shoulder soft tissue coverage using LDMF has also been reported in several studies.^3^, ^15^, ^16^ Nevertheless, the functional reconstruction of the shoulder musculature using the pedicled LDMF has only been described to our knowledge in two case reports.^17^, ^18^ Paprottka *et al*. used the innervated LDMF to reconstruct only the medial part of the deltoideus muscle after radical tumor resection, without additional bony reconstruction. In our case after the injury, the LD lost its humeral insertion and therefore was rendered functionless. Dosari *et al*. also presented in a case report a patient with deltoid deficient shoulder after a history of gunshot injury, who was treated with a RSA after Latissimus dorsi flap and could show a successful treatment with an active abduction of 45° and external rotation of 20°.^18^ Nevertheless, as opposed to our case, the viability of the remaining native anterior deltoid (as evident by preoperative needle EMG) was shown preoperatively and the surgical intervention was done in a two‐stage approach. The uniqueness of our case was the reconstruction of the whole deltoideus muscle function and therefore restoration of the shoulder movement by inserting the LD tendon on the humerus shaft and fixating the LD origin on the neck musculature, on top of the revisional shoulder arthroplasty, while maintaining the blood and nervous supply intact.

The result after 1 year showed a very satisfying aesthetical result, with a natural shoulder contour and no residual pain. Considering the shoulder mobility, the patient showed an appropriate arm flexion, which enabled him to bring the hand in a functional position. On the other hand, the rather limited arm abduction could be due to the nonphysiological biomechanics of the hemiarthroplasty in the absence of the rotator cuff. However, the implantation of a reverse shoulder arthroplasty would have been accompanied by a disproportional risk of implant dislocation with the potential damage of LDMF. The 40° abduction could nevertheless enable him to use the arm freely for light everyday activities.

### Conclusions

Our case showed the successful reconstruction of a complexly destroyed shoulder joint. The interdisciplinary approach used a novel transposition of the innervated LDMF to rebuild motion and contour and a shoulder arthroplasty to replace the joint. Finally, function and aesthetic could be restored.

## Author Contributions

Olimpiu Bota conceptualized, wrote, and edited the manuscript together with Jörg Nowotny, Adrian Dragu, Florian Bönke, Eric Tille and Feras Taqatqeh helped gathering the data and revised the final version of the manuscript. All authors read and approved the manuscript.
